# Benchmarking short and long read polishing tools for nanopore assemblies: achieving near-perfect genomes for outbreak isolates

**DOI:** 10.1186/s12864-024-10582-x

**Published:** 2024-07-08

**Authors:** Tu Luan, Seth Commichaux, Maria Hoffmann, Victor Jayeola, Jae Hee Jang, Mihai Pop, Hugh Rand, Yan Luo

**Affiliations:** 1 Department of Computer Science, University of Maryland, College Park, MD 20742 USA; 2 Center for Food Safety and Applied Nutrition, Food and Drug Administration, Laurel, MD 20708 USA; 3 Center for Food Safety and Applied Nutrition, Food and Drug Administration, College Park, MD 20740 USA

**Keywords:** Nanopore sequencing, Long read sequencing, Assembly polishing, Benchmarking, Bacterial genomics, *Salmonella*, Food poisoning outbreaks, Source tracking investigations

## Abstract

**Background:**

Oxford Nanopore provides high throughput sequencing platforms able to reconstruct complete bacterial genomes with 99.95% accuracy. However, even small levels of error can obscure the phylogenetic relationships between closely related isolates. Polishing tools have been developed to correct these errors, but it is uncertain if they obtain the accuracy needed for the high-resolution source tracking of foodborne illness outbreaks.

**Results:**

We tested 132 combinations of assembly and short- and long-read polishing tools to assess their accuracy for reconstructing the genome sequences of 15 highly similar *Salmonella enterica* serovar Newport isolates from a 2020 onion outbreak. While long-read polishing alone improved accuracy, near perfect accuracy (99.9999% accuracy or ~ 5 nucleotide errors across the 4.8 Mbp genome, excluding low confidence regions) was only obtained by pipelines that combined both long- and short-read polishing tools. Notably, medaka was a more accurate and efficient long-read polisher than Racon. Among short-read polishers, NextPolish showed the highest accuracy, but Pilon, Polypolish, and POLCA performed similarly. Among the 5 best performing pipelines, polishing with medaka followed by NextPolish was the most common combination. Importantly, the order of polishing tools mattered i.e., using less accurate tools after more accurate ones introduced errors. Indels in homopolymers and repetitive regions, where the short reads could not be uniquely mapped, remained the most challenging errors to correct.

**Conclusions:**

Short reads are still needed to correct errors in nanopore sequenced assemblies to obtain the accuracy required for source tracking investigations. Our granular assessment of the performance of the polishing pipelines allowed us to suggest best practices for tool users and areas for improvement for tool developers.

**Supplementary Information:**

The online version contains supplementary material available at 10.1186/s12864-024-10582-x.

## Background

Whole genome sequencing (WGS) provides actionable information in areas as diverse as precision medicine, the tracking of plant pathogens in crops, climate-induced microbiome shifts, detecting the evolution of viral variants, and the tracing of nosocomial infections [1–5]. WGS has also revolutionized the bioinformatic strain typing and source attribution of foodborne bacterial pathogens, largely facilitated by rapid response networks such as GenomeTrakr [6] and PulseNet [7]. These programs primarily rely on the high accuracy (sequencing error rate ≤ 0.1% [8]) and throughput of short-read sequencing platforms such as the Illumina Miseq and NextSeq. Accurate strain typing of bacterial pathogens is typically accomplished using short reads for single nucleotide polymorphism (SNP) analysis or the assembled genomes for core-genome or whole-genome multi-locus sequence typing (cgMLST, or wgMLST, respectively) analysis. Despite providing high resolution, these methods do not fully utilize the genomic information, such as the synteny and colocation of coregulated features, intragenic regions, mobile elements (e.g., phages, transposons, insertion sequences, plasmids), and repetitive sequences [9–11]. This is because the short reads (300 bp or shorter) cannot span most bacterial genome repeats, resulting in fragmented assemblies and collapsed repeat regions [12, 13]. As a result, clinically relevant features, such as the *Salmonella* pathogenicity islands and plasmids, may not be completely reconstructed from the raw reads [14].

Pacific Biosciences (PacBio) and Oxford Nanopore Technologies (ONT) provide long-read sequencing platforms that routinely produce reads of 10 kbp or longer. The long reads can span most of the bacterial genome repeats and, therefore, allow the reconstruction of complete bacterial chromosomes and plasmids. Complete genomes enhance the resolution of phylogenetic analyses and the analysis of horizontal gene transfer networks, and provide higher quality data for the discovery of novel genotypes associated with clinically relevant phenotypes (e.g., antimicrobial resistance, virulence, persistence) [15, 16]. Over the past decade, long-read sequencing platforms have become increasingly competitive with short-read sequencing in terms of cost, throughput, and sequencing accuracy. As a result, long-read sequencing has led to many breakthroughs such as: the complete sequencing of the human genome [17], the recovery of complete bacterial genomes from metagenomes [18], and real-time tracking of epidemics and pandemics [19, 20].

When comparing the PacBio and ONT platforms, PacBio HiFi sequencing can produce highly accurate reads that can reconstruct highly accurate (> 99.999% [21]) and nearly complete bacterial genomes from pure cultures and metagenomes. However, the sequencer is expensive, not portable, and has a relatively high cost per sequenced base [22–24]. In contrast, some ONT platforms like the MinIon are portable and provide real-time sequencing, are cost-effective, and have a relatively simpler library preparation, but the reconstructed bacterial genomes are less accurate (~ 99.95%) [25]. For context, a 5 Mbp bacterial genome with 99.95% accuracy still contains 2,500 nucleotide errors. These errors can obscure the estimation of phylogenetic relationships between outbreak isolates, which may only differ by a few nucleotides [13, 26–28]. Many of the errors in the nanopore assemblies are inherited from the reads which often have a sequencing error rate between 5 and 15%. Most of the sequencing errors are insertions or deletions (indels) associated with repetitive genomic regions, such as homopolymers, short repeats, or regions with high GC content, and are caused by the variable translocation speed of the DNA through the nanopore [29].

Many computational tools for error correction (or “polishing”) have been developed to address the high number of errors in nanopore assemblies. A common polishing strategy involves aligning the reads back to the assembly using tools such as Minimap2 [30], BWA-MEM [31], or Bowtie2 [32]. The read pile-up is then used to assess the evidence for each nucleotide in the assembly, enabling the identification of errors. Both short and long reads can be used for polishing, each providing different strengths and weaknesses. For example, long reads are more error-prone but there is less ambiguity about where they align in an assembly because they can span genomic repeats. In contrast, short reads are more accurate and have a different error profile than nanopore reads, but might fail to correct errors, and even introduce new errors in repetitive genomic regions [33, 34]. Currently, pipelines that utilize both long- and short- read polishing provide the best improvement in assembly accuracy. However, such approaches also increase the cost, labor, and complexity of the polishing process [13].

In the context of foodborne pathogen genomics, it has been shown that polished nanopore assemblies can have nearly concordant SNP and cgMLST profiles with the corresponding short-read assemblies [13, 35, 36]. However, errors that persist in the nanopore assemblies after polishing can sometimes result in incorrect phylogenetic clustering and the misidentification of open reading frames, potentially affecting gene predictions [14, 33]. It is uncertain how accurately, comprehensively, and consistently the errors that persist after polishing can be identified—a challenge that needs to be addressed before nanopore assemblies can be routinely and reliably used for source tracking analyses.

**Fig. 1:**
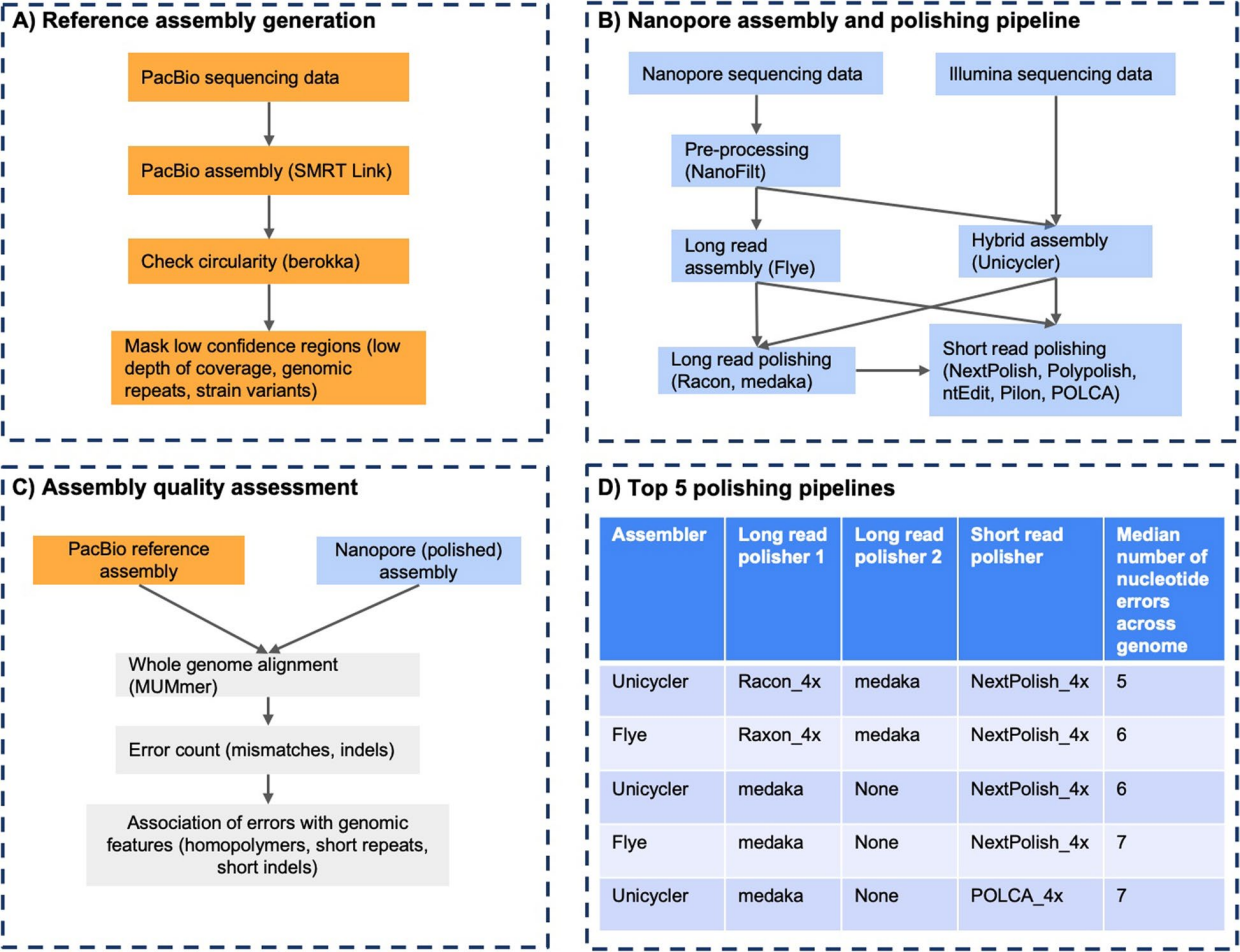
The workflow of the analysis in this study. **A** Creation of the reference genomes involved PacBio HiFi long-read sequencing the 15 *Salmonella* Newport isolates, assembling the reads, and masking low confidence regions in the assemblies. **B** The 15 isolates were also short-read sequenced with an Illumina MiSeq and long-read sequenced with a Nanopore GridIon. The long reads were used to generate the Flye assemblies, and both the short and long reads were used to create the Unicycler hybrid assemblies. Then long and short read polishing was performed on the assemblies in 132 combinations. **C** The quality of the assemblies was assessed by aligning the polished assemblies to the reference genomes and counting the number of errors in the alignments. Then the errors were associated with genomic features such as homopolymers and short repeats if they were adjacent to the error. **D** The 5 most accurate polishing pipelines produced assemblies that were near-perfect i.e., they contained a median of 5 to 7 nucleotide errors across the genome length, which was ~ 4.85 Mbp long

With this study, we aimed to provide a granular assessment of the accuracy of polished nanopore assemblies, by combining state-of-the-art assembly and short- and long-read polishing tools (Table 1) in various pipelines (Fig. 1). The performance and error profile of each pipeline were assessed by counting the number and types of errors and their association with genomic features. The results of our study highlight the strengths and weaknesses in currently available assembly and polishing tools, and allow us to recommend best practices and areas for future research.

**Table 1:** Summary of the polishing tools used for this study. The bolded category under the “Read type used for polishing” column indicates the read type used with the tool for this study

Tool	Year published	Read type used for polishing	Types of errors tool can fix	Known to sometimes introduce new errors
medaka	2018 (was made public but is not published)	**long**	SNPs, small indels	Yes
Racon	2017	short, **long**	SNPs, small indels	Yes
NextPolish	2020	**short**	SNPs, small indels	Yes
Polypolish	2022	**short**	SNPs, small indels	Yes
POLCA	2020	**short**	SNPs, small indels	Yes
Pilon	2014	**short**, long	SNPs, small indels, large indels, misassemblies	Yes
ntEdit	2019	short	SNPs, small indels	Yes

## Results

### Summary of the sequencing data

While long-read sequencing platforms generated substantially fewer reads compared to the MiSeq (see Supplementary File 1), they achieved much higher coverage of the *Salmonella* Newport (lineage III) genomes due to their longer read lengths. The median coverage depths were 372X, 223X, and 82X for the HiFi, GridIon, and MiSeq reads, respectively. Read lengths also differed substantially, with HiFi reads averaging 13 kbp, GridIon reads 2 kbp, and MiSeq reads a much shorter 251 bp. In terms of sequencing accuracy, the HiFi reads displayed the highest alignment accuracy to the reference genomes (99.8%), followed by MiSeq (99.6%) and GridIon (91.7%).

### Summary of the PacBio reference genomes

The HiFi reads were assembled into closed, circular chromosomes and plasmids, providing the reference genomes for our analysis (Supplementary Table 1). The number of plasmids per isolate ranged from 1 to 9. The chromosomes of the isolates were highly similar, with a median length of 4.85 Mbp and a median number of 4,535 genes. The 4,429 core chromosomal genes had identical synteny, and the total number of pairwise chromosomal differences ranged from 96 to 87,625 nucleotides. The nucleotide differences were mainly explained by the loss or gain of prophages.

### Repetitive genomic regions in the reference genomes

Homopolymers and short genomic repeats can cause nanopore sequencing errors that are difficult to correct with polishing tools. These problematic regions covered a median of 15.9% (homopolymers) and 21.1% (short genomic repeats) of the reference genomes (Supplementary Table 1). Further, approximately 3.5% of the reference genomes contained repetitive genomic regions (where the short reads multi-mapped) where errors are challenging to correct for short-read polishers. Notably, some of these regions contained genes associated with virulence in humans (Supplementary Table 2) [37–41].

### Masking low confidence regions in the reference genomes

We used the reference genomes to evaluate the quality of the polished nanopore assemblies. As such, it was important to mask regions in the reference genomes where we had low confidence if a nucleotide difference was due to an error in the reference genome or in the nanopore assembly. Low confidence regions were defined as windows (longer than 5 bp) where the PacBio reads aligned to their corresponding reference genome:

1. With a low depth of coverage (less than 40X).
2. With a low median MapQ read alignment score (less than 40), indicative of repetitive genomic regions.
3. Indicated polymorphic loci, suggesting the presence of multiple strains.

Details about the masking procedures and justifications are provided in the Methods section and Supplementary Table 3. The masked regions primarily corresponded to repetitive sequences (68%) or low coverage areas (31%), with total lengths ranging from 23 bp to 92 kbp per assembly. Notably, a ~ 1 kbp inverted segment, crucial for flagellar phase variation virulence gene expression [42], was masked in ten assemblies. Additionally, two assemblies with extensive masking likely harbored low-abundance plasmids or population-variable phages.

### Large-scale differences between the reference genomes and the Flye and Unicycler assemblies

All the chromosomes were reconstructed as single contigs in the reference genomes as well as the Flye, and Unicycler assemblies, except for one Unicycler assembly where the chromosome was broken into two contigs. One Unicycler assembly also had 8 contigs that appeared to be duplicated fragments of the completely assembled chromosomes—these were removed from downstream analyses. The Unicycler assemblies were closer in length to the references than the Flye assemblies, with a median absolute difference of 609 bp and 16,800 bp, respectively. This was because the Flye assemblies contained many insertion errors and the contigs were sometimes over-circular i.e., the start and end of the assembly might overlap, typically by a read length or less. Out of 15 isolates, there were 5 PacBio, 2 Flye, and 1 Unicycler assemblies where a single plasmid (ranging in size from 2 to 88 kbp) did not assemble compared to the other assemblies of the same isolate (Supplementary Table 4). We confirmed in each case that the plasmids were not present in the reads.

### Comparison between the Unicycler and Flye assemblies

**Fig. 2:**
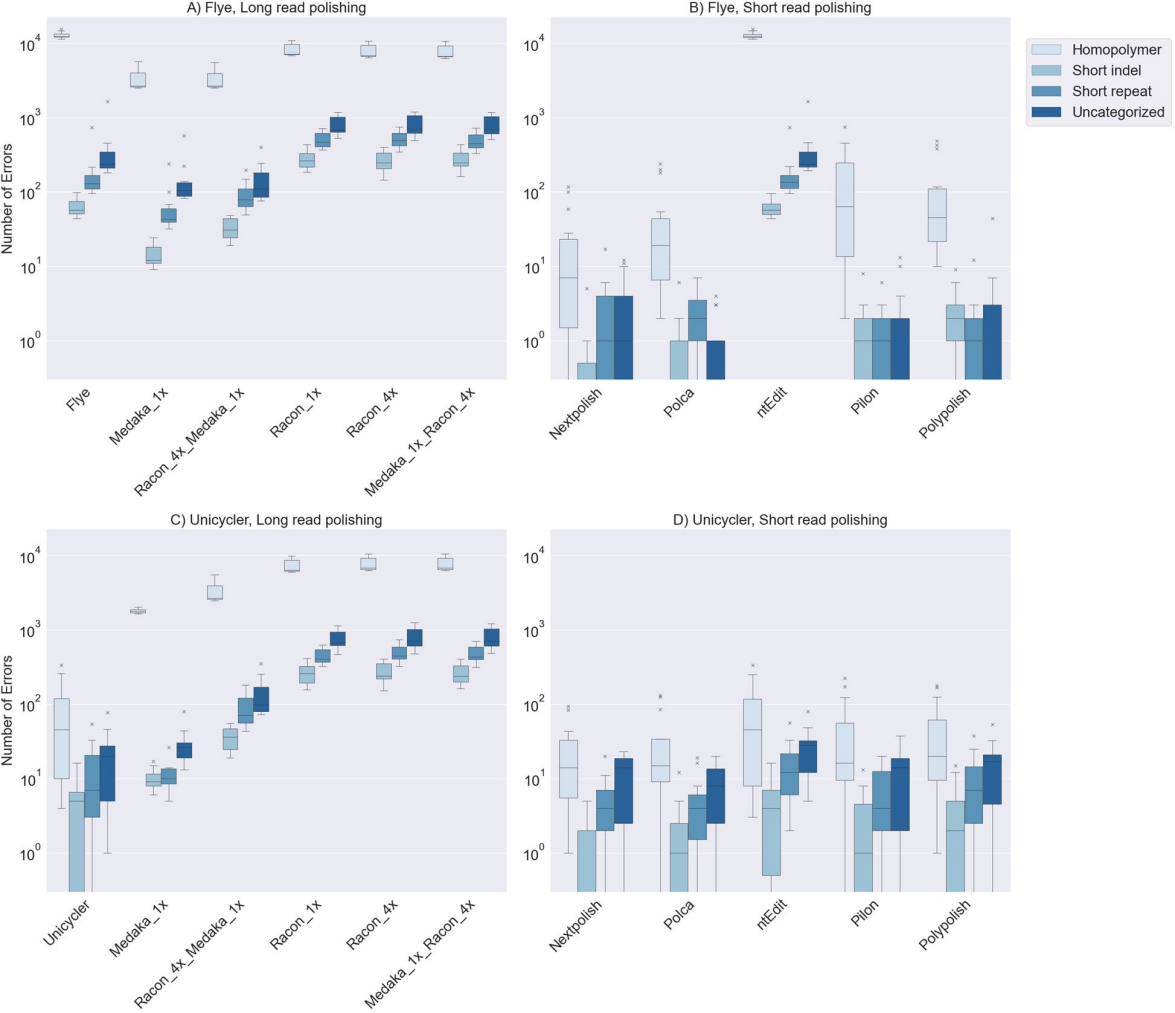
The genomic features associated with the errors in the assemblies polished by long or short reads only. The genomic features associated with the errors in the long-read polished **A** Flye and **C **Unicycler assemblies. The genomic features associated with the errors in the short-read polished **B **Flye and **D **Unicycler assemblies. The uncategorized errors could not be associated with a known genomic feature. Plot was created with matplotlib in Python

The Unicycler assemblies were substantially more accurate than the Flye assemblies (Table 2) with a median accuracy of 99.998% (75 nucleotide errors) and 99.7% (13,557 nucleotide errors), respectively. This is expected because Unicycler uses both the accurate short reads and the error-prone nanopore reads to build hybrid assemblies. In contrast, Flye only uses the nanopore reads for assembly. The indel-to-mismatch ratio was much higher for the Flye assemblies (47:1) than the Unicycler assemblies (4:1). The errors in the Flye and Unicycler assemblies were primarily associated with homopolymers, 96% and 67%, respectively (Fig. 2). The majority of the remaining errors in the Unicycler assemblers were associated with short repeats (11%) and uncharacterized genomic features (18%).

**Table 2:** Summary of error types, error locations, and associated genomic features in the polished nanopore assemblies

				Error location (median values)		Errors associated with genomic features (median values)				Percent errors in repetitive genomic regions where short reads do not map uniquely
Tool Type	Pipeline	Total errors (median)	Indel to mismatch ratio	Chromosome	Plasmid	Short Repeats	Short indels	Homopolymers	Unknown	
Assembler	Flye	13,575	47:1	13,273	361	130	57	12,318	234	3%
	Unicycler	75	4:1	75	1	7	5	45	20	33%
Long-read polisher used last	Racon	9517	41:1	9323	251	442	248	6883	691	3%
	medaka	2963	39:1	3609	125	79	34	3326	132	3%
Short-read polisher	NextPolish	10	2:1	6	1	2	0	4	1	43%
	POLCA	21	6:1	18	1	2	1	10	2	33%
	Polypolish	44	10:1	34	3	3	2	24	2	27%
	Pilon	52	17:1	132	6	3	3	110	6	10%
	ntEdit	8808	38:1	8452	183	283	119	6317	473	3%
Top 5 performing pipelines	Unicycler_Racon_4x_medaka_NextPolish_4x	5	1:1	4	1	2	0	1	1	30%
	Flye_Racon_4x_medaka_NextPolish_4x	6	1:1	4	0	1	0	3	1	31%
	Unicycler_medaka_NextPolish_4x	6	1:1	4	1	2	0	1	1	32%
	Flye_medaka_NextPolish_4x	7	1:1	4	1	1	0	3	1	30%
	Unicycler_medaka_POLCA_4x	7	22:1	5	1	0	0	4	0	28%

### Errors after long-read polishing

Thousands of nucleotide errors remained, even after the Flye and Unicycler assemblies were long-read polished with medaka and/or Racon. The effectiveness of the polishing tools depended heavily on the tool(s) selected, their order in the pipeline, and the initial assembler used (Table 2, Fig. 2).

#### Unicycler with medaka excelled

Unicycler assemblies polished with medaka achieved the highest accuracy (median of 99.96%, 1,916 nucleotide errors). medaka consistently outperformed Racon, regardless of whether it was used alone or combined (median errors: medaka—2,963, Racon—9,517). Notably, both tools exhibited a similar indel-to-mismatch ratio (40:1) and errors primarily occurred within homopolymers (~ 86%).

#### Polishing order matters

When the polishers were combined, the order they were used in the pipeline significantly impacted the final result. Polishing with medaka after Racon generally reduced errors, while the opposite order (Racon after medaka) introduced new errors (Table 3).

**Table 3:** The impact of polishing iterations and long-read polishing tools (medaka or Racon) on short-read polishing accuracy. Negative values indicate the median number of nucleotide errors corrected, while positive values (bolded) represent the median number of nucleotide errors introduced during polishing

Assembler	Short-read polisher	1 versus 4 iterations of polishing	Long-read polishing with Racon	Long-read polishing with medaka
Flye	NextPolish	0	0	-3
Unicycler	NextPolish	0	-28	-32
Flye	POLCA	-10	**8**	-17
Unicycler	POLCA	-5	-11	-30
Flye	Polypolish	-6	-1	-50
Unicycler	Polypolish	-2	**19**	-39
Flye	Pilon	-500	-127	-385
Unicycler	Pilon	-327	**319**	**31**
Flye	ntEdit	0	-3925	-10,499
Unicycler	ntEdit	0	**9185**	**2423**

#### The impact of assembler

The assembler used also played a crucial role (Table 3). Long-read polishing corrected thousands of nucleotide errors in Flye assemblies, with medaka correcting errors associated with homopolymers, short repeats, short indels, and unknown genomic features. Conversely, Racon mainly corrected homopolymer-associated errors and either did not correct errors associated with the other three categories or introduced new errors. For Unicycler assemblies, all polishing combinations tended to introduce errors (sometimes thousands) across all four categories, with the sole exception of medaka when used alone, which occasionally corrected errors within short repeats and uncharacterized genomic features.

### Errors after short-read polishing

This section examines the effectiveness of the five short-read polishing tools (POLCA, Pilon, ntEdit, Polypolish, and NextPolish) on Flye and Unicycler assemblies, both directly and after long-read polishing.

#### NextPolish excelled

Overall, NextPolish emerged as the most accurate tool, only leaving a median of 10 nucleotide errors (Table 2). Other tools like POLCA, Polypolish, and Pilon (with four iterations) performed well too, with medians ranging from 21 to 52 nucleotide errors. Notably, ntEdit was the least effective, with many more errors (median: 8,808).

#### Mismatches were corrected at a higher rate than indels

All tools tended to correct more mismatches than indels, with indel-to-mismatch ratios varying between 2:1 (NextPolish) and 38:1 (ntEdit). While NextPolish had the fewest indel errors (median: 9), Polypolish had the fewest mismatch errors (median: 1).

#### More iterations are not necessarily better

Increasing the number of polishing iterations from one to four generally had minimal impact on error correction (Table 3). For instance, additional rounds with POLCA, Polypolish, NextPolish, and ntEdit only corrected a median of 7.5, 2.5, 0, and 0 errors respectively. The improvements for POLCA and Polypolish were mainly driven by correcting short indels for POLCA, and short indels and short repeats for Polypolish. Unlike the other tools, Pilon exhibited a substantial improvement with more iterations (median: 438.5 additional nucleotide errors corrected), primarily associated with homopolymers (Supplementary Fig. 1). We further tested Pilon with up to eight iterations, but observed minimal additional error correction after five rounds.

#### Impact of long-read polishing

Incorporating long-read polishing before short-read polishing generally enhanced assembly accuracy, with medaka proving more effective than Racon (Table 3). However, as mentioned earlier, long-read polishers often introduced new errors in Unicycler assemblies, leading to lower accuracy when combined with short-read polishers compared to using short-read polishers alone. Conversely, short-read polishing directly applied to Flye assemblies (without long-read polishing) greatly improved accuracy, while minimal improvement was seen for the already highly accurate Unicycler assemblies (Fig. 2).

#### Shared errors among short-read polishers

**Fig. 3:**
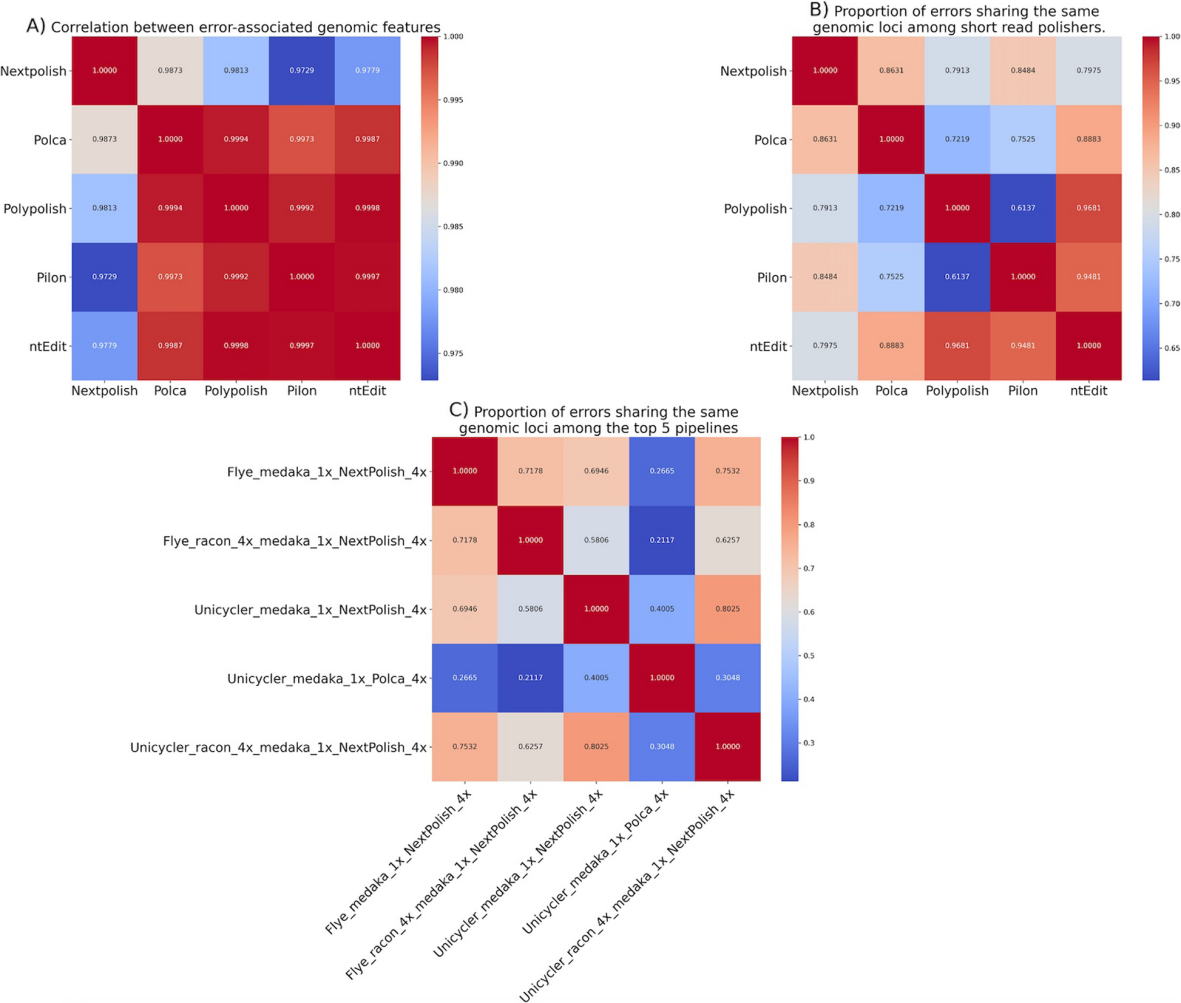
**A** Heatmap Illustrating the correlation matrix of mean errors across samples by the associated genomic features (i.e., homopolymers, short repeats, short indels, uncharacterized features) of the short-read polishers. **B** Heatmap illustrating the proportion of shared error loci for the short-read polishers. **C** Heatmap illustrating the proportion of shared error loci among the top five pipelines. Plot was created with matplotlib in Python

We compared the overlap in error locations between short-read polishers by dividing the number of shared loci by the minimum number of errors observed for the compared tools (Fig. 3). In general, a high proportion (0.61 to 0.97) of errors occurred at the same locations in the short-read polished assemblies. ntEdit had the highest similarity (mean = 0.97) with the other tools. This is likely because ntEdit corrects very few errors in the initial assemblies compared to other tools and, thus, has a higher chance of sharing errors with them. Conversely, Polypolish showed the least similarity to any other tool (mean = 0.77).

#### Error distribution across genomic features

There was no significant enrichment of errors on plasmids compared to chromosomes and the proportions of errors associated with various genomic features (homopolymers, short repeats, short indels, uncharacterized) were quite similar across the short-read polishers. NextPolish showed the lowest correlation (mean correlation of 0.98) with other tools (Fig. 3A). For all short-read polishers, most errors were associated with homopolymers, ranging from 63% (NextPolish) to 87% (Pilon and ntEdit). Notably, POLCA, Polypolish, and NextPolish showed enrichment of errors in A and T homopolymers, while ntEdit had more errors in G and C homopolymers (Supplementary Table 5). The second most common association was with uncharacterized features, ranging between 7% (Pilon and ntEdit) and 20% (NextPolish).

#### Short-read mapping and long indels

The percentage of errors falling within short-read multi-mapped regions (~ 3.5% of the reference genomes) ranged from 43% for NextPolish to 3% for ntEdit (Table 2), with the most accurate tools correcting more errors outside these regions. Long indels (20 to 100 bp) were rarely associated with errors. Only seven polished assemblies from isolate CFSAN110829 had a single long indel each. Notably, none of the long indel sequences were found in either the short or long reads, suggesting they were artifacts generated by the assembly and/or polishing tools.

### The five most accurate pipelines

The five most accurate pipelines produced assemblies with a median of 5 to 7 nucleotide errors (Table 2). Two pipelines utilized the Flye assembler, while three employed Unicycler. Notably, three pipelines underwent long-read polishing with medaka, and two received long-read polishing with four iterations of Racon followed by medaka. Finally, four pipelines were short-read polished with NextPolish, and one with POLCA.

The percentage of errors with shared loci (Fig. 3C) was higher within the NextPolish pipelines (58% to 80%) compared to those shared between NextPolish and the POLCA pipelines (21% to 44%).

**Fig. 4:**
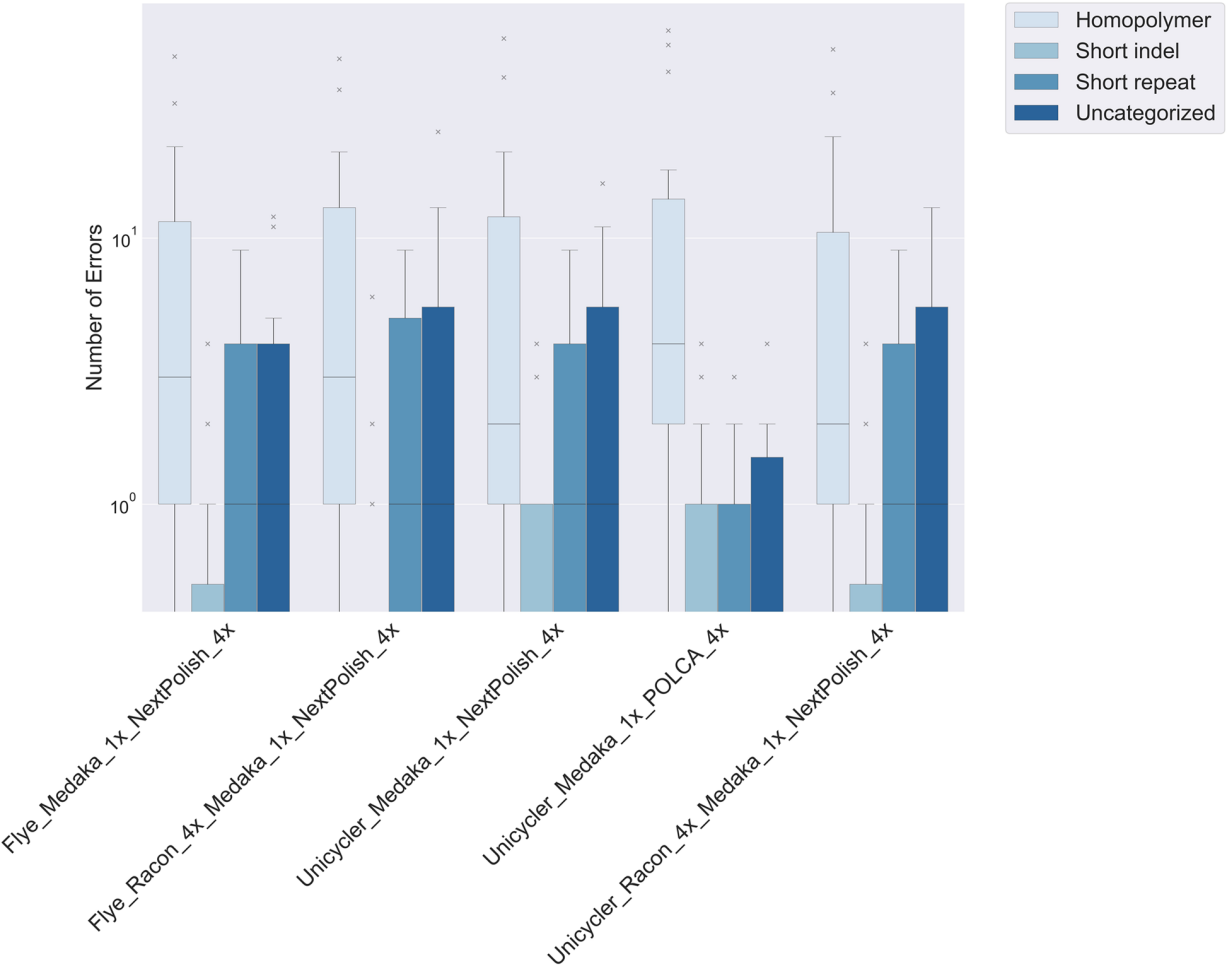
The genomic features associated with the errors in the five best performing polishing pipelines. Each of these pipelines incorporated long-read polishing followed by short-read polishing. The uncategorized errors could not be associated with a known genomic feature. Plot was created with matplotlib in Python

The POLCA-polished assemblies exhibited a high indel-to-mismatch ratio (21:1), with errors primarily associated with homopolymers (86%). Conversely, the NextPolish pipelines displayed a roughly 1:1 indel-to-mismatch ratio. Approximately 61%, 14%, 4%, and 21% of these errors were linked to homopolymers, short repeats, short indels, and uncharacterized features, respectively (Fig. 4).

A closer examination of the uncharacterized errors revealed that features, like homopolymers, were frequently present within 10 bp windows of the error, rather than directly adjacent to or covering it. Recategorizing the uncharacterized features within these windows yielded a revised distribution of errors: 76% associated with homopolymers, 18% with short repeats, 4% with short indels, and 2% with uncharacterized features. Notably, homopolymer-associated errors had a median length of 6 nucleotides and exhibited a bias towards A and T nucleotides (83% and 89% for NextPolish and POLCA, respectively), instead of C and G.

### Performance when combining tools with complementary error profiles

Our previous analyses suggested that combining short-read polishers with different error profiles might result in higher accuracy. We selected NextPolish and Polypolish for this experiment because they differed substantially in the location of errors, the genomic features associated with the errors, and the error type (indels vs. mismatches). For each previously run NextPolish pipeline we ran it in combination with Polypolish, using one or four iterations. Similarly, we ran each previously run Polypolish pipeline in combination with NextPolish.

Overall, none of the pipelines sequentially combining NextPolish and Polypolish outperformed the five most accurate pipelines from our previous analysis. Rather, the most accurate combination of NextPolish and Polypolish performed worse 33% of the time or tied the five most accurate pipelines 66% of the time. Nonetheless, using NextPolish after Polypolish always corrected additional errors, whereas using Polypolish after NextPolish never corrected additional errors.

### Runtime and memory usage

#### Memory usage

The only tool that used more memory than the limit of 36 GB of RAM was the assembler Flye. Flye exceeded the 36 GB RAM limit during assembly of the largest read sets (CFSAN110836 and CFSAN110838) and on sample CFSAN110833 which was smaller than other read sets that assembled successfully. Randomly downsampling these read sets to 2 × 10^5^ reads successfully resolved the memory issue for Flye.

### Runtime analysis

Supplementary Table 6 summarizes the runtime details for all tools used in this study. The total runtime for the pipelines (including assembly, short-read polishing, and long-read polishing) varied considerably from 8 to 677 min.

#### Assemblers

Flye had lower runtimes than Unicycler (median runtime: 27 min for Flye vs. 210 min for Unicycler). This is likely because Unicycler builds hybrid assemblies and, by default, incorporates long-read polishing with Racon and short-read polishing with Pilon, whereas Flye only uses the long reads to build an assembly with no additional polishing.

#### Long-read polishers

When incorporated, long-read polishing substantially increased runtime—accounted for a median of 59% and 14% of the runtime when combined with Flye or Unicycler for assembly, respectively. Medaka was faster than Racon (median runtime: 5 min for medaka vs. 11 min for Racon with 1 iteration, and 44 min for Racon with 4 iterations). Combining Racon and medaka had an additive effect on runtime regardless of order.

#### Short-read polishers

Amongst short-read polishers, ntEdit was by far the fastest, with a median of 7 s to finish one iteration or 31 s for four iterations. The speed of ntEdit is due to its use of rapid exact k-mer matching instead of the more computationally expensive read alignment used by other tools. For other short-read polishers, a single iteration took between 3 and 7 min, and four iterations took between 12 and 25 min. In each case, Polypolish had the fastest runtimes and Pilon the slowest.

#### Runtime of the most accurate pipelines

The five most accurate pipelines had median runtimes ranging from 49 to 277 min. Given that long-read polishing can be a consuming step, pipelines that only used medaka were the fastest.

## Discussion

### Complete genomes and outbreak insights

Although short-read sequencing is the current state-of-the-art public health response for the bioinformatic source tracking of foodborne illness outbreaks, nanopore sequencing provided valuable insights into the outbreak that would have been difficult to obtain with short-read data alone. While short-read SNP analysis suggested minimal differences between isolates (median pairwise SNP difference = 0) [43], the complete genomes revealed significant nucleotide variations (up to 90 kbp in just the chromosomes). The complete genomes also revealed that the core genes of the isolates had identical synteny, an independent means of assessing their close phylogenetic relatedness [15, 16]. Additionally, long reads enabled the identification of virulence-associated genes and large phage duplications missed by short-read sequencing (Supplementary Table 2). Furthermore, long reads revealed the location of large strain variants (e.g., a ~ 1 kbp inverted segment associated with virulence [42]), highlighting the limitations of assembly algorithms in representing multiple strains. Many assembly algorithms only reconstruct the genome of the most abundant strain or a mosaic of the strains, when multiple strains are present in a read set. Although strain variation can be difficult to represent in assembly graphs and contigs, tool developers should still provide this information directly or in a variant file format.

### Limitations of long-read sequencing

Despite the advantages of long-read sequencing, we observed instances where plasmids were missing from the PacBio or nanopore sequencing data or where the assemblers failed to reconstruct them. This has been observed before and highlights the need for improved sequencing protocols and assembly algorithms to preserve plasmid information [44]. Furthermore, even long reads could not fully resolve the genome sequence in some regions (low confidence regions). These regions were excluded when counting errors in the nanopore assemblies, emphasizing the need for careful scrutiny and development of methods to resolve or flag these problematic areas.

### Long-reads are not sufficiently accurate for outbreak tracking

The complete nanopore assemblies, without further polishing, were not sufficiently accurate for source tracking of foodborne illness outbreaks, where a small number of genomic differences (0–20 SNPs) can be crucial for regulatory decisions [13]. Our nanopore data accuracy increased from 91.7% for reads to 99.7% for Flye assemblies and 99.96% after long-read polishing (corresponding to ≥ 1,900 nucleotide errors). Notably, these results were obtained with the ONT R9.4.1 flow cell, which has lower accuracy compared to the newer R10.4 cell with sequencing accuracy of 95% or higher [45]. Future work should explore newer ONT platforms and how assembly tools can better utilize read information to reduce reliance on long-read polishing.

### Impact of short-read polishing

Consistent with previous work, incorporating short-read polishing substantially increased the median accuracy of the nanopore assemblies, reaching up to 99.9999% [13]. The assemblies reconstructed with the five most accurate pipelines were practically identical to the reference genomes given that they were within the range of accuracy expected for HiFi sequenced assemblies [21]. However, only 21% to 80% of the remaining nucleotide differences occurred in the same locations, indicating that some were errors. Notably, these pipelines did not rely on a single set of tools. Both Flye and Unicycler were used, along with medaka and Racon (though the order of long-read polishers mattered – the best pipelines used medaka alone or medaka after Racon). For short-read polishing, four out of the five best pipelines used NextPolish, with one using POLCA.

### Performance of short-read polishing tools

Across all tested combinations, NextPolish was the most accurate short-read polisher, followed closely by POLCA, Polypolish, and Pilon. Other studies have shown these tools perform similarly, but the best performing tool often depended on the dataset characteristics (species content, GC content, amount of repetitive genomic sequences) [46, 47]. One advantage we observed for NextPolish was that it often achieved its highest accuracy with just one iteration, whereas other tools benefited from additional runs.

Pilon was notable for needing at least four iterations of polishing to obtain its best accuracy.

In contrast to other tools and consistent with benchmarks, ntEdit showed very poor accuracy [13, 47]. This is likely because ntEdit permutes k-mers from the assembly until they match k-mers in the read set for error correction, instead of using read alignments like the other tools. This approach is problematic because the short, permuted k-mers are more likely to occur multiple times in a genome than the reads, increasing the likelihood a permuted k-mer will match a spurious genomic location.

### Error distribution and short-read multi-mapped regions

Similar to previous studies, most errors in the nanopore assemblies were systematic and not randomly distributed [29]. Indels associated with homopolymers were the most challenging to correct for all polishing tools. Notably, sequencing errors associated with homopolymers also affect Illumina and PacBio HiFi platforms [48, 49], making it difficult to completely polish errors or generate a perfect reference. Errors associated with short repeats and short indels were less common and easier to correct. At a broader scale, we observed a disproportionate number of errors in short-read multi-mapped regions, even for the most accurate tools. Only Polypolish explicitly handles errors in these regions [46], but its performance was unexpectedly worse than NextPolish and POLCA. This highlights the ongoing challenge of multi-mapped reads for short-read polishing tools.

### Short-read polishing tool nuances

Although the short-read polishing tools showed similar abilities for correcting errors associated with specific features, they differed in detail. Some tools were better at correcting specific homopolymer types (A/T vs. C/G), and they also differed in the locations of errors and the indel-to-mismatch ratio. Variations in the indel-to-mismatch ratio can impact phylogenetic analyses. For example, the CFSAN SNP Pipeline excludes many indels from its analysis [50]. Therefore, a tool like Polypolish, with more indel errors and fewer mismatches, might be preferable for such analyses compared to NextPolish.

The ability of tools to correct different error types suggests that combining multiple polishing tools might improve accuracy. We tested this idea using combinations of NextPolish and Polypolish (tools with different error-correcting properties) in a single pipeline. While our analysis did not show improvement, other studies have reported higher accuracies when combining short-read polishing tools [46]. This highlights the potential for such approaches. At least, the differential error-correcting abilities suggest opportunities to develop new tools that leverage the strengths and weaknesses of existing tools.

### Importance of tool order in pipelines

A crucial observation from our study for tool users and developers was that using less accurate tools after more accurate ones often introduced errors. This was seen with:

- Racon or medaka for long-read polishing of Unicycler assemblies
- Long-read polishing with Racon after medaka
- Short-read polishing with Polypolish after NextPolish

### Balancing accuracy, efficiency, and user friendliness

Another consideration when combining tools into pipelines is the trade-off between accuracy, computational efficiency, and user-friendliness. For example, Unicycler is a standalone tool with high accuracy. However, Flye assemblies (initially less accurate) supplemented with any short-read polishing tool (except ntEdit) could often match or exceed Unicycler's accuracy (Fig. 2) with considerably faster runtimes, though with higher memory usage [51–53]. Similarly, among the five most accurate pipelines, the one using Flye, medaka, and four iterations of NextPolish had the fastest runtime (50 min), while the second fastest took nearly twice as long (93 min). Similar accuracy with an even shorter runtime could be achieved by running the same pipeline with just one iteration of NextPolish (median errors increase from 5 to 10, but median runtime decreases from 57 to 46 min).

## Conclusion

Our analysis revealed that complete genome assemblies were achievable with nanopore reads, but polishing was essential for high accuracy. While long-read polishing improved accuracy, near perfect accuracy (99.9999% accuracy or ~ 5 nucleotide errors across the entire genome, excluding the low confidence regions) was only obtained with pipelines that combined both long and short-read polishing tools. Notably, medaka was a more accurate and efficient long-read polisher compared to Racon. Among short-read polishers, NextPolish showed the highest accuracy, but other tools like Pilon, Polypolish, and POLCA performed similarly. Among the 5 best performing pipelines, long-read polishing with medaka followed by short-read polishing with NextPolish was the most common high-performing combination. Importantly, the order of polishing tools mattered i.e., using less accurate tools after more accurate ones introduced errors. Indels in homopolymers and repetitive regions, where the short reads could not be uniquely mapped, remained the most challenging errors to correct.

Our case study of a set of highly similar genomes from a foodborne illness outbreak provides an analytical framework for future investigations of diverse genomes. Our granular approach went beyond basic assembly statistics and highlights that 99.9% accurate assemblies can no longer be considered highly accurate, especially for applications like outbreak source tracking where small variations (like 5 SNPs) can be crucial. As large genome sequencing collections continue to grow (e.g., the NCBI Pathogen Detection Database exceeding 1 million isolates), ongoing tool development and careful analysis, as demonstrated here, will be critical. Ultimately, high-accuracy long-read assemblies will empower researchers to delve deeper into biological questions.

## Methods

### Working definitions for repetitive genomic regions

#### Homopolymer

A sequence with the same nucleotide repeated 3 or more times, e.g., AAAA, GGGGG.

#### Short repeat

A sequence with 2 or 3 nucleotides that consecutively repeat two or more times, e.g., AGAG, ACTACT, excluding homopolymers.

#### Multi-mapped regions and reads

Repetitive regions of an assembly where short reads align equally well, i.e., with the same alignment score.

#### Low confidence regions

Regions of a reference genome where the aligned PacBio Hifi reads indicated genomic repeats, low depth of coverage, or strain variants.

### Data and sequencing

The dataset (Supplementary File 1) consisted of 15 clinical isolates that had been collected during the 2020 onion outbreak associated with *Salmonella enterica* serovar Newport (lineage III) [54]. The 15 isolates were selected because they were the 15 clinical isolates (out of all 1,728 clinical isolates from the outbreak) that together provided the maximal coverage of the genes in the pangenome (74.4%). The 15 isolates were sequenced on both the Oxford Nanopore Technologies (ONT) and Pacific Biosciences (PacBio) long-read platforms so that closed genomes could be reconstructed. The Illumina Miseq short reads were downloaded from the NCBI SRA database (accessions in Supplementary File 1).

The bacteria were grown overnight in tryptic soy broth (TSB) at 37˚C and genomic DNA was extracted using the Maxwell RSC cultured cell DNA kit (Promega, Madison, WI) following the manufacturer’s protocols. The DNA was used to construct libraries for the GridION (Oxford Nanopore Technologies, Oxford, UK) using the rapid sequencing kit RBK004, which was run on a MIN106D flow cell (R9.4.1) for 48 h according to the manufacturer’s instructions.

The multiplexed microbial SMRTbell libraries were prepared using the SMRTbell Template Prep Kit 2.0 according to PacBio protocol “Preparing Multiplexed Microbial Libraries Using SMRTbell Express Template Prep Kit 2.0” (PacBio, Menlo Park, CA, November 2021). The multiplexed SMRTbell library was then sequenced on a PacBio Sequel IIe sequencer (PacBio, Menlo Park, CA) using Binding Kit 2.2 and Sequel II sequencing Kit 2.0 on one SMRT cell 8 M (PacBio, Menlo Park, CA), with 30 h collection time.

### The PacBio reference genomes

The PacBio HiFi reads were assembled using the Microbial Genome Analysis pipeline within SMRT Link (v11.0) [55]. The circularity of the assembled chromosomes and plasmids was confirmed with berokka [56]. These assemblies were used as the references for our analysis and referred to as the reference genomes. Gene predictions were made with Prokka (v1.14.5) [57] and plasmid identification was performed with Platon (v1.6) [58]. The pangenome of the reference genomes was estimated with Roary (v3.12.0) [59]. The pairwise number of SNPs was identified with the Mummer package (v4.0.0) [60]. All non-overlapping homopolymers and short repeats in the reference genomes were identified using *genome_repeat_content.py* (see Availability of Data and Materials).

The accuracy of the short and long reads was assessed by aligning them to their corresponding reference genome with MiniMap2 (v2.1) [30] and then counting the number of mismatches and indels per read alignment and the median across all the aligned reads. Command-line BLAST was used to identify regions of the reference genomes with no corresponding sequence in the short-read assemblies—the reads were downloaded from NCBI's SRA database and assembled with SPAdes (v3.13.0) [61]. Online BLASTX was then used on the NCBI website, using the NCBI nr database, to identify protein-coding genes in the unmapped regions of the reference genome.

### Exclusion of unreliable regions in the reference genomes

A pipeline was created to exclude genomic regions with low quality or ambiguity from the reference genomes (referred to as low confidence regions), as they were considered unreliable for identifying errors in the polished nanopore assemblies. The amount of each genome that was masked is listed in Supplementary Table 3. The first step of the pipeline mapped the PacBio HiFi reads to the corresponding reference genome with MiniMap2. Samtools was used to convert the read alignment file from SAM to BAM format and to create the pileup of reads so that all bases from all mapped reads could be counted for each locus in the assembly. Two Python scripts, *summarize_mpileup.py* and *unusual_genomic_loci.py* (see Data Availability), were used to parse the read pileups and to identify low confidence regions.

Three metrics were used to identify low confidence regions in the reference genomes. 1) A mean MapQ score below 40, suggesting a genomic repeat where the PacBio HiFi reads aligned with equal alignment scores. 2) A depth of coverage less than 40X, signaling diminished confidence in the consensus base call of the assembly, particularly with respect to the presence of strain variants. Although it has been shown that PacBio HiFi reads with 28X coverage can obtain equivalent SNP-calling accuracy as short reads with 30X coverage [62], we erred on the side of caution considering the benchmarking nature of this study and the potential presence of multiple strains. 3) If there was evidence for multiple nucleotide variants. Here, this was defined as the presence of one or more variants that were ≥ 40% as abundant as the most abundant variant.

### Description of the tools used for assembly and polishing

#### Assemblers

Two assemblers, Flye [25] and Unicycler [63], were selected for our study because each had thousands of citations and recent benchmarking studies had shown that they consistently produced highly contiguous and accurate assemblies compared to other assembly tools [13, 64–66].

Flye uses the long reads to form error-prone disjointigs (concatenations of multiple disjoint genomic segments). The disjointigs are then concatenated to construct a repeat graph. Repeats that are bridged by aligned reads are directly resolved, whereas unbridged repeats are resolved using information about their copy number and the alignments of the long reads to the repeat graph.

Unicycler was originally created as a hybrid assembler (uses both short and long reads), but was recently updated to facilitate long-read assembly too [63, 67]. For our experiments, Unicycler was used as a hybrid assembler. In this mode, Unicycler first assembles the short reads with SPAdes [61]. The multiplicity of contigs is then determined based upon read depth and graph connectivity. Short and long read bridges are used to resolve repeats and to simplify the assembly graph based upon the bridging quality scores. The resulting assembly is polished with Pilon as described below under the Short-read polishing section [68].

#### Long-read polishing tools

To polish the nanopore assemblies with long reads, we used two popular state-of-the-art tools, medaka [69] and Racon [70] (Table 1).

Medaka is a tool developed by Oxford Nanopore, and the algorithm it employs has not yet been published in a peer-reviewed venue. Based on the information on its GitHub page, the medaka algorithm vectorizes the pileup of the long-read alignments to the assembly by the count of each nucleotide and passes it into a pre-trained model based on a long short-term memory (LSTM) recurrent neural network (RNN). The consensus is output as the prediction from the model. The pre-trained models were specifically built for use with Flye assemblies, however, medaka also allows users to train their own models.

Racon first aligns the long reads to the assembly, then applies multiple filters to remove low-quality read alignments. The assembled contigs and mapped reads are then split into non-overlapping windows where the consensus is derived by constructing a partial order alignment graph (POA) using simple instruction multiple data (SIMD) acceleration.

#### Short-read polishing tools

To polish the nanopore assemblies with the short reads, we used five popular state-of-the-art tools: Pilon [68], NextPolish [71], Polypolish [46], POLCA [47], and ntEdit [72] (Table 1).

Pilon uses the consensus of the read alignments to correct single base errors and indels that are shorter than a read length. Pilon attempts to identify and resolve larger indels and misassemblies based upon anomalous read coverage and read mapping patterns (e.g., high proportion of soft-clipped alignments, mate pairs that do not map with correct library size). If there are large gaps in the assembly, Pilon will attempt to reassemble the reads across that region.

POLCA utilizes Freebayes [73], a variant detection software, to identify SNPs and indels from the read alignments to the assembly. When an alternative allele (substitution or indel) is observed and the count of the alternative allele is twice more than the count of the original allele from the input assembly, POLCA will regard the assembly as having a putative error and correct it to the alternative allele with the highest count.

NextPolish uses a two-step approach for polishing. The first step constructs a k-mer score chain based upon the read alignment pileup. This is done by choosing the nucleotide at each locus that has the best score based on the preceding base and the count of 3-mers containing both bases. The corrected sequence is then found using a traceback procedure. The second step uses a k-mer count module to correct regions of the assembly where the reads mapped with low quality or low depth of coverage. The k-mers covering the flagged regions as well as the most frequent k-mers are used for correction. An additional round of applying the k-mer score chain (first step) is then used to correct these regions after the depth of coverage has been adjusted.

Polypolish also relies on the consensus of short-read alignments to the long-read assembly for polishing. However, unlike the tools mentioned previously, it considers all possible alignment locations of a read to the assembly to provide increased coverage of genomic repeats to better resolve them. To account for indel errors associated with genomic homopolymers, Polypolish trims any homopolymer, plus an extra base, at the end of an aligned read or, if there are none, the last two bases. Polypolish builds the consensus sequence by calculating depth of coverage at each position—uniquely mapped reads contribute a single unit of coverage, and multi-mapped reads contribute a fractional unit of coverage (the reciprocal of their alignment count). By default, corrections are made when over 50% of the reads, with a minimum of 5X read coverage, disagree with the assembly.

Unlike the previously mentioned tools that analyze the pileup of short-read alignments to the long-read assembly, ntEdit is a k-mer based approach. ntEdit first constructs a Bloom filter for the k-mers extracted from the short reads. If k-mers extracted from the assembly are not present in the Bloom filter they are flagged as errors. Assembly k-mers flagged as errors are permuted by changing each base to one of the 3 alternative bases, starting from the 3' end, and then queried against the Bloom filter. Permuted k-mers with sufficient evidence within the Bloom filter are used to correct the assembly, otherwise they undergo a second round of permutations using insertions and deletions. This process continues until a correction is made with sufficient support, or all possible edits have been exhausted.

### Pipeline to assemble the nanopore long reads and polish the assemblies

We first filtered out nanopore reads with a read quality score less than 10 using NanoFilt (v2.8.0) [74]. The Flye (v2.9-b1768) assemblies were built using the filtered long reads, and the Unicycler (v0.5.0) assemblies were built using the filtered long reads and the short reads, combined.

Flye did not successfully generate outputs for samples CFSAN110836 and CFSAN110838 using 36 GB of RAM and 8 cores, even when using Flye’s parameter for downsampling to reduce memory usage for high coverage data. For that reason, the two samples were manually downsampled to a count of 200 k reads for all analyses in our study for consistency. We performed a bootstrapping analysis, randomly resampled 200 k reads 10 times for each isolate, to verify that the selection of reads did not affect the completeness or synteny of the resulting Flye assemblies.

The assemblies were polished with long reads using medaka (v1.7.2) and Racon (v1.4.12) either separately or in combination (medaka followed by Racon or vice versa). In each pipeline, Racon was run using one or four iterations to assess how the number of iterations, a user tunable parameter, affected assembly quality. Medaka was always run with one iteration because that was the common practice found in the literature [75, 76].

For short-read polishing, we employed POLCA (Masurca 4.0.5), Pilon (v1.24), ntEdit (v1.3.5), Polypolish (v0.5.0), and NextPolish (v1.4.0). The short-read polishing tools were utilized either directly on the initial Unicycler and Flye assemblies or on the assemblies following long-read polishing. Each short-read polishing tool was run with one or four iterations. Additionally, Pilon was run with 5 to 8 iterations—this was because Pilon was the only short-read polisher that showed a substantial increase in accuracy from one to four iterations. For Pilon, Polypolish and NextPolish, the short reads were aligned to the draft assemblies with BWA (v0.7.17-r1188) [31] before being passed to the polishers. For POLCA and ntEdit, the short reads were directly passed to the polishers without pre-processing.

In total, we tested 132 pipelines per isolate, combining different assembly and polishing tools, including: the assemblies with no polishing, the assemblies with long-read polishing only, the assemblies with short-read polishing only, and the assemblies with long-read polishing followed by short-read polishing. The resulting assemblies from each polishing pipeline were trimmed and circularized using berokka.

### Comparing the unpolished and polished nanopore assemblies to the reference genomes

Both the unpolished and polished nanopore assemblies were compared to their respective reference genomes using NUCmer from the MUMmer package (v4.0.0) [60]. The MUMmer program “show-snps” was used to identify mismatches and indels in genomic regions where there were no ambiguous alignments (show-snps—× 10 -C). The total number of errors was normalized by multiplying by the total assembly length and dividing by the total alignment length.

$$Normalized\;Total\;Errors=Total\;Errors\;\times\frac{Total\;Assembly\;Length}{Total\;Alignment\;Length}$$

A Python script, *parse_nucmer_alignments.py* (see Data Availability), was used to identify the genomic features associated with the errors: indels, short repeats, and homopolymers. These genomic features could either flank or span across the error site.

### Runtime analysis

All jobs were run with 8 cores on a high-performance computing cluster (3.0 GHz AMD® EPYC® 7313 Processor) with a memory ceiling of 36 GB RAM. To assess and document the computational runtime for the polishing pipeline, the Linux "time" command was used, and the actual elapsed time among all concurrently active threads was recorded.

### Supplementary Information

Supplementary Material 1.

Supplementary Material 2.

## Data Availability

All the Illumina MiSeq, PacBio HiFi, and Oxford Nanopore GridIon sequencing data can be found in the NCBI SRA database under the following isolate identifiers: CFSAN110825-CFSAN110829, CFSAN110832-CFSAN110838, CFSAN110902, CFSAN112116, and CFSAN112117. The scripts used for this analysis and to run the assembly and polishing pipelines can be found here https://github.com/tluan/CARTS21_scripts.
