# Supplementary material for: Benchmarking short and long read polishing tools for nanopore assemblies: achieving near-perfect genomes for outbreak isolates
Source: BMC Genomics. 2024 Jul 8;25:679. doi: 10.1186/s12864-024-10582-x (PMC11232133; doi:10.1186/s12864-024-10582-x)
Supplement: Supplementary file 2 — Supplementary Material 2. [file 12864_2024_10582_MOESM2_ESM.docx]

# SUPPLEMENTARY TABLES

Supplementary Table 1. Summary statistics about the annotated reference assemblies.

| Sample | Number of contigs | Total assembly length (bp) | Number of genes | Chromosome length (bp) | Number of chromosomal genes | Number of plasmids | Number of plasmid genes | Total plasmid length (bp) | Total length of homopolymers (bp) | Total length of short repeats (bp) | Bases of genome where short reads do not map uniquely |
| --- | --- | --- | --- | --- | --- | --- | --- | --- | --- | --- | --- |
| CFSAN110825 | 2 | 4844012 | 4524 | 4770496 | 4449 | 1 | 75 | 73516 | 771448 | 1022780 | 169541 |
| CFSAN110826 | 10 | 5104881 | 4846 | 4813027 | 4506 | 9 | 340 | 291854 | 814486 | 1061229 | 287013 |
| CFSAN110827 | 2 | 4844037 | 4527 | 4770505 | 4451 | 1 | 76 | 73532 | 771191 | 1022938 | 165138 |
| CFSAN110828 | 2 | 4885243 | 4581 | 4811731 | 4504 | 1 | 77 | 73512 | 777945 | 1030921 | 239910 |
| CFSAN110829 | 6 | 5008212 | 4726 | 4769576 | 4453 | 5 | 273 | 238636 | 797579 | 1046546 | 191544 |
| CFSAN110832 | 3 | 4898252 | 4602 | 4769863 | 4450 | 2 | 152 | 128389 | 780753 | 1030157 | 180941 |
| CFSAN110833 | 3 | 4875473 | 4569 | 4770503 | 4448 | 2 | 121 | 104970 | 777481 | 1027480 | 216639 |
| CFSAN110834 | 3 | 4932061 | 4620 | 4770516 | 4448 | 2 | 172 | 161545 | 785174 | 1036677 | 166489 |
| CFSAN110835 | 2 | 4843421 | 4532 | 4769911 | 4455 | 1 | 77 | 73510 | 771365 | 1022570 | 166833 |
| CFSAN110836 | 2 | 4843721 | 4535 | 4770214 | 4460 | 1 | 75 | 73507 | 771221 | 1022726 | 160010 |
| CFSAN110837 | 3 | 4877683 | 4573 | 4770511 | 4447 | 2 | 126 | 107172 | 777407 | 1027859 | 162894 |
| CFSAN110838 | 2 | 4842936 | 4528 | 4769422 | 4452 | 1 | 76 | 73514 | 771130 | 1022565 | 172495 |
| CFSAN110902 | 2 | 4844148 | 4522 | 4770571 | 4446 | 1 | 76 | 73577 | 771452 | 1022845 | 170143 |
| CFSAN112116 | 3 | 4848102 | 4521 | 4770525 | 4443 | 2 | 78 | 77577 | 771953 | 1023365 | 170219 |
| CFSAN112117 | 2 | 4843998 | 4518 | 4770486 | 4443 | 1 | 75 | 73512 | 771248 | 1022674 | 149259 |

Supplementary Table 2. List of virulence-associated genomic features that occurred in regions that did not assemble in the short read assemblies.

| **Virulence gene/feature** | **Function** |
| --- | --- |
| Heme ABC exporter | Aids in scavenging heme from the human host [37] |
| Phage-associated recT recombinase | Increases the efficiency of incorporating DNA sequences into bacterial chromosomes and plasmids [38, 39] |
| IS3 elements |  |
| Cell wall-associated hydrolase | Aids in biofilm formation and cell wall modifications to evade the host immune response [40] |
| Sodium ion-translocating decarboxylase | Involved with energy generation and the maintenance of pH homeostasis under conditions of low pH or high concentrations of organic acids such as in the gastrointestinal tract [41] |

Supplementary Table 3. Summary of regions that were masked in the reference assemblies.

| Isolate | Regions (>5 nt) with low depth of coverage  (< 40X) | Regions (>5 nt) with low median MapQ read alignment score  (< 40) | Polymorphic loci (nt) | Whole genome alignment error (nt) | Chromosome-associated loci (nt) | Plasmid-associated loci (nt) | Total nucleotides excluded | Main biological features associated with excluded regions |
| --- | --- | --- | --- | --- | --- | --- | --- | --- |
| CFSAN110825 | 42 | 966 | 100 | 0 | 1,107 | 1 | 1,108 | Polymorphic loci, hin-recombinase-associated inversion |
| CFSAN110826 | 22,545 | 69,718 | 58 | 0 | 71,297 | 21,024 | 92,375 | Polymorphic loci, hin-recombinase-associated inversion, low abundance plasmids, duplicated phage |
| CFSAN110827 | 15 | 966 | 34 | 0 | 997 | 18 | 1,015 | Polymorphic loci, hin-recombinase-associated inversion |
| CFSAN110828 | 26,937 | 36,626 | 32 | 0 | 63,591 | 4 | 63,595 | Polymorphic loci, hin-recombinase-associated inversion, duplicated phage |
| CFSAN110829 | 2,044 | 966 | 61 | 0 | 1,016 | 2,055 | 3,071 | Polymorphic loci, hin-recombinase-associated inversion |
| CFSAN110832 | 10 | 966 | 32 | 0 | 992 | 16 | 1,008 | Polymorphic loci, hin-recombinase-associated inversion |
| CFSAN110833 | 77 | 0 | 32 | 0 | 37 | 72 | 109 | Polymorphic loci |
| CFSAN110834 | 0 | 0 | 23 | 0 | 21 | 2 | 23 | Polymorphic loci |
| CFSAN110835 | 0 | 966 | 42 | 0 | 1,007 | 1 | 1,008 | Polymorphic loci, hin-recombinase-associated inversion |
| CFSAN110836 | 11 | 0 | 50 | 75 | 130 | 2 | 132 | Polymorphic loci |
| CFSAN110837 | 100 | 0 | 32 | 0 | 26 | 106 | 132 | Polymorphic loci |
| CFSAN110838 | 0 | 717 | 1,001 | 0 | 1,024 | 226 | 1,250 | Polymorphic loci, hin-recombinase-associated inversion |
| CFSAN110902 | 106 | 966 | 139 | 0 | 1,087 | 123 | 1,210 | Polymorphic loci, hin-recombinase-associated inversion |
| CFSAN112116 | 9 | 0 | 286 | 0 | 76 | 219 | 295 | Polymorphic loci |
| CFSAN112117 | 104 | 966 | 21 | 0 | 1,090 | 1 | 1,091 | Polymorphic loci, hin-recombinase-associated inversion |

Supplementary Table 4. Comparison of the total assembly lengths and number of assembled plasmids for the Flye, Unicycler, and reference assemblies.

| Isolate | Total assembly length | | | Plasmids (length of unassembled plasmid) | | |
| --- | --- | --- | --- | --- | --- | --- |
|  | Reference | Flye | Unicycler | Reference | Flye | Unicycler |
| CFSAN110825 | 4844012 | 4828957 | 4843730 | 1 | 1 | 1 |
| CFSAN110826 | 5104881 | 5107349 | 5032289 | 9 (2,403 nt) | 10 | 9 (73,221 nt) |
| CFSAN110827 | 4844037 | 4862009 | 4877748 | 1 (33,548 nt) | 2 | 2 |
| CFSAN110828 | 4885243 | 4829831 | 4844051 | 1 | 1 | 1 |
| CFSAN110829 | 5008212 | 4995218 | 5008821 | 5 | 4 (4,087 nt) | 5 |
| CFSAN110832 | 4898252 | 4886408 | 4898911 | 2 | 2 | 2 |
| CFSAN110833 | 4875473 | 4858963 | 4875370 | 2 | 2 | 2 |
| CFSAN110834 | 4932061 | 4992071 | 4932079 | 2 | 2 | 2 |
| CFSAN110835 | 4843421 | 4826591 | 4843779 | 1 | 1 | 1 |
| CFSAN110836 | 4843721 | 4830660 | 4844038 | 1 | 1 | 1 |
| CFSAN110837 | 4877683 | 4898323 | 4877608 | 2 | 2 | 2 |
| CFSAN110838 | 4842936 | 4916500 | 4948521 | 1 (85,985 nt) | 2 | 2 |
| CFSAN110902 | 4844148 | 4830775 | 4843794 | 1 | 1 | 1 |
| CFSAN112116 | 4848102 | 4917085 | 4934503 | 2 (86,149 nt) | 2 (4,064 nt) | 3 |
| CFSAN112117 | 4843998 | 4918181 | 4931668 | 1 (87,759 nt) | 2 | 2 |

Supplementary Table 5. Percentage of homopolymer-associated errors by homopolymer type for the last assembly or polishing tool used in a pipeline.

| **Homopolymer type** | **Pilon** | **POLCA** | **Polypolish** | **NextPolish** | **ntEdit** | **Flye** | **Unicycler** | **Reference genomes** |
| --- | --- | --- | --- | --- | --- | --- | --- | --- |
| **A** | 34% | 42% | 39% | 41% | 31% | 33% | 38% | 33% |
| **C** | 16% | 9% | 12% | 9% | 19% | 17% | 12% | 17% |
| **G** | 16% | 7% | 10% | 6% | 19% | 17% | 13% | 17% |
| **T** | 34% | 42% | 39% | 43% | 31% | 33% | 38% | 33% |

Supplementary Table 6. Runtimes for the tools and the best performing pipelines used in this study. All jobs were run with 8 cores on a high-performance computing cluster (3.0GHz AMD® EPYC® 7313 Processor) with a memory ceiling of 36 GB RAM.

| Tool Type | Pipeline (number of iterations of polishing) | Minimum  (minute:second) | Median  (minute:second) | Maximum  (minute:second) |
| --- | --- | --- | --- | --- |
| Assembler | Flye | 4:20 | 26:52 | 105:49 |
|  | Unicycler | 114:42 | 210:04 | 487:13 |
| Long-read polisher | Racon_1x | 3:13 | 11:18 | 39:42 |
|  | Racon_4x | 12:48 | 44:50 | 155:50 |
|  | medaka_1x | 3:43 | 5:14 | 10:52 |
|  | medaka_1x_Racon_4x | 17:05 | 49:00 | 160:54 |
|  | Racon_4x_medaka_1x | 17:06 | 48:31 | 161:26 |
| Short-read polisher | NextPolish_1x | 1:41 | 4:10 | 9:12 |
|  | NextPolish_4x | 6:35 | 16:27 | 36:36 |
|  | ntEdit_1x | 0:05 | 0:07 | 0:17 |
|  | ntEdit_4x | 0:22 | 0:31 | 1:09 |
|  | Pilon_1x | 2:26 | 7:06 | 19:42 |
|  | Pilon_4x | 8:56 | 25:16 | 63:36 |
|  | POLCA_1x | 1:18 | 4:15 | 14:51 |
|  | POLCA_4x | 4:37 | 16:02 | 46:57 |
|  | Polypolish_1x | 1:05 | 3:20 | 10:04 |
|  | Polypolish_4x | 4:05 | 12:20 | 39:54 |
| Top 5 performing pipelines | Unicycler_Racon_4x_medaka_NextPolish_4x | 159:19 | 277:04 | 668:47 |
|  | Flye_Racon_4x_medaka_NextPolish_4x | 44:15 | 92:46 | 287:59 |
|  | Unicycler_medaka_NextPolish_4x | 132:33 | 232:28 | 515:26 |
|  | Flye_medaka_NextPolish_4x | 30:30 | 49:25 | 133:45 |
|  | Unicycler_medaka_POLCA_4x | 129:33 | 232:46 | 511:43 |

# SUPPLEMENTARY FIGURES


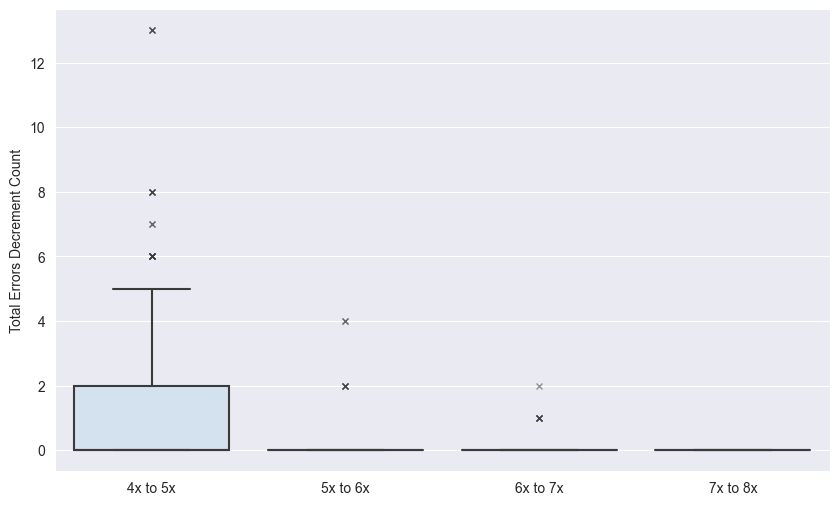


Supplementary Figure 1. The effect of additional iterations of polishing on the accuracy of Pilon. Pilon was the only short read polisher where increasing the iterations of polishing from one to four substantially decreased the number of errors. The boxplot shows the change in total errors when running Pilon with 5 to 8 iterations of polishing.
